# microGLYMPH: a conceptual translational roadmap for microdialysis‑based assessment of CSF–interstitial solute exchange in acquired brain injury

**DOI:** 10.1186/s13054-026-06063-0

**Published:** 2026-05-05

**Authors:** Nagesh C. Shanbhag, Chisomo Zimphango, Nils Hecht, Bryn A. Martin, Christos Panotopoulos, Elisa Gouvea Bogossian, Fabio S. Taccone, Andres M. Rubiano, Peter J. Hutchinson, Niklas Marklund, Jefferson W. Chen, Peter Vajkoczy

**Affiliations:** 1 Meditech Foundation, Valle del Cauca, Cali, 760031 Colombia; 2 Division of Neurosurgery, Department of Clinical Neurosciences, University of Cambridge, Cambridge, CB2 0QQ UK; 3 Department of Neurosurgery, Charite Universitatsmedizin, Universität Berlin, Humboldt-Universität zu Berlin, Berlin Institute of Health, Berlin, 10117 Germany; 4 Department of Chemical and Biological Engineering, University of Idaho, Moscow, 83843 ID USA; 5 Flux Neuroscience, LLC, Troy, ID USA; 6 Department of Neurosurgery, Mediterraneo Hospital, Glyfada, 16675 Greece; 7 Department of Intensive Care, Hôpital Universitaire de Bruxelles (HUB), Universite Libre de Bruxelles (ULB), Brussels, 1050 Belgium; 8 Neuroscience Institute, Neurotrauma Group, El Bosque University, Bogotá, 110121 Colombia; 9 Department of Clinical Sciences Lund, Neurosurgery, Lund University, Skane University Hospital, Lund, 22185 Sweden; 10 Department of Neurosurgical Surgery, University of California, Irvine Medical Center, Orange, 92868 CA USA

## Abstract

The glymphatic system facilitates cerebrospinal fluid (CSF)–interstitial fluid exchange and plays a key role in solute clearance and neurophysiological homeostasis. While dysfunction of this system has been shown in traumatic brain injury, stroke, meningitis, idiopathic normal pressure hydrocephalus and neurodegenerative diseases, direct measurement of glymphatic transport in humans remains elusive. We propose microGLYMPH as a translational, hypothesis-generating framework that combines established clinical cerebral microdialysis with controlled CSF tracer administration via existing clinical access routes, including an external ventricular drain, cisternal access during surgery, or lumbar intrathecal injection when clinically justified. The aim is to obtain time-resolved regional tracer profiles in microdialysate and to interpret these alongside arousal state, intracranial dynamics, and, where available, complementary imaging, thereby providing an indirect measure of CSF–interstitial exchange kinetics and peripheral tracer appearance. We further define the key design, analytical and practical limitations that must be resolved before the approach can extend beyond exploratory use, notably catheter-adjacent effects, blood–brain barrier disruption, drainage practices, and the intrinsically focal nature of microdialysis. microGLYMPH is therefore intended as a staged roadmap for first-in-human feasibility studies and subsequent hypothesis-driven investigations of neurofluid solute transport after acute brain injury.

## Introduction

The glymphatic system facilitates extravascular cerebrospinal fluid (CSF) flow into the brain parenchyma, aiding in interstitial fluid (ISF) solute clearance, especially during non-rapid eye movement (NREM) sleep and under reduced arousal states while modulated by cardiorespiratory impulses, arterial pulsatility and brain vasomotion [1–3]. Glymphatic dysfunction has been demonstrated in traumatic brain injury (TBI), ischemic stroke, meningitis and subarachnoid hemorrhage as well as in neurodegenerative diseases, and anoxic injuries [1–7]. Recent evidence suggests that glymphatic dysfunction precedes early brain edema, while neuroinflammation and impairment in the neurofluid dynamics (comprising of arteriovenous blood, CSF and ISF compartments) impedes the subsequent glymphatic drainage as observed in rodent models of brain injuries [4, 5]. Following TBI, evidence suggests that glymphatic clearance is impaired, potentially contributing to chronic neuroinflammation, tau aggregation, and long-term neurodegeneration [8]. TBI impairs the glymphatic function including solute drainage along the cervical lymphatic network in murine models [4].

Human glymphatic studies have primarily relied on imaging (e.g., structural, diffusion-weighted, and dynamic contrast-enhanced, DCE-MRI using intrathecal (lumbar subarachnoid space, SAS) or intravenous gadolinium-based contrast agents, GBCAs such as gadobutrol) or postmortem histopathological analyses, without direct quantification of solute movement between CSF and ISF [9–11]. Consequently, the dynamics of this dysfunction, its persistence over time, and its relationship to clinical outcomes are poorly understood. CSF imaging would allow detailed characterization of glymphatic injury and recovery trajectories after TBI, offering opportunities for biomarker discovery and therapeutic targeting.

Cerebral microdialysis (CMD), a relatively widely used tool in neurocritical care, enables semi-continuous sampling of interstitial molecules including brain-derived proteins [12], and may also serve as a pragmatic readout of regional tracer appearance in interstitial fluid when tracers are introduced into a CSF compartment. In this Perspective, we outline a staged translational concept in which clinically used tracers (with explicit attention to regulatory status) are administered via established CSF access routes and quantified in microdialysate to generate testable CSF‑to‑interstitium kinetic hypotheses. By combining this approach with the administration of traceable solutes such as gadobutrol, iodinated contrast (Iohexol), or novel putative agents (metabolically inert) into a CSF compartment (i.e., intraventricular, intracisternal or lumbar SAS), we can estimate time‑resolved CSF–interstitial exchange kinetics at the probe region, overcoming limitations of imaging alone (Table 1). Furthermore, a direct assessment of transport pathways (CSF-to-brain-to-periphery) can be achieved by assessing the solute level in body fluids as a measure of brain solute clearance. If validated, this approach could contribute to experimental stratification, longitudinal monitoring and intervention testing in selected contexts and assess therapeutic interventions aimed at restoring or enhancing glymphatic clearance. Longitudinal studies, enabled by the safety profile of macrocyclic GBCAs like gadobutrol [13, 14], and iodinated contrast agents could elucidate how glymphatic dysfunction evolves over time and interacts with disease mechanisms, while serving as a prognostication biomarker to predict acute as well as long-term outcomes. Repeated measurements in the same individual would also allow for tracking glymphatic changes over disease progression or therapeutic interventions (e.g., hypertonic saline, decompressive craniectomy, craniotomy, cisternostomy). Together, such studies could illuminate a previously underappreciated mechanism of secondary injury and recovery, opening new avenues for clinical translation.

**Table 1:** Clinically used CSF contrast agents

Contrast agent(s)	Molecular weight (kDa)	Half-life (h)	Standard imaging modalities	Typical clinical intrathecal status	Quantification in micro-dialysate & body fluids	Clinical & ethical feasibility
**GBCAs** Gadobutrol (Gadavist)	0.6	1.8	MRI	Off‑label	ICP-MS	Yes
**Iodinated agents** Iohexol (Omnipaque)	0.82	1.5	CT	Labelled intrathecal (at specific concentrations/ uses)	HPLC-UV X-ray fluorometry LC-MS/MS	Yes
**Radiotracers** ^99^Tc-DTPA ^111^In-DTPA ^64^Cu-DOTA ^68^Ga-DOTA	0.48 0.5 1.49 1.5	6 68 12.7 0.68	SPECT-CT SPECT-CT PET-CT PET-CT	Variable / protocol‑specific	Scintillation counter (radioactivity)	Problematic
**Fluorescent tracer(s)** Sodium fluorescein	0.33	0.26	Intraoperative fluoroscopy	Context dependent	Spectrophotometry	Yes
	**Scope for novel CSF tracer repurposing**,** development and optimizations** • Metabolically inert molecules • High molecular weight (MW) agents • Co-injection of low- and high MW agents					

CSF, cerebrospinal fluid; GBCAs, gadolinium-based contrast agents; MRI, magnetic resonance imaging; ICP-MS, inductively coupled plasma mass spectrometry; CT, computed tomography; HPLC-UV, high performance liquid chromatography-ultraviolet; LC-MS/MS, liquid chromatography with tandem mass spectrometry; DTPA, diethylene triamine penta acetic acid; DOTA, dodecanetetraacetic acid; SPECT-CT, single photon emission tomography-computed tomography; PET, positron emission tomography

## Preliminary data and feasibility

### Pre-clinical investigations

Preclinical assessment of CSF–ISF transport relies on tracer-based labeling of the CSF space (cisterna magna, intraventricular or intrathecal) using size-calibrated fluorescent [e.g., dextran or bovine serum albumin-conjugated Alexa647], GBCAs, or radiolabeled molecules (e.g., ^99m^Tc-DTPA), coupled with in vivo imaging and ex vivo histology to quantify solute movement through perivascular and lymphatic pathways [1, 2, 15–19]. Typical implementations combine: (i) intracisternal or intraventricular injection of relevant tracers, (ii) two-photon or stereomicroscopic in vivo imaging of perivascular and parenchymal influx, (iii) DCE-MRI or DCE-Radionuclide-CT for tracer distribution within the CNS and glymphatic pathways, and (iv) terminal whole brain-spinal cord tissue sampling and cervical lymph node assays to map efflux. This multimodal pipeline provides spatially and temporally resolved evidence of CSF influx along peri-arterial routes and efflux along perivenous counterparts including perineural sheath spaces of cranio-spinal nerves, and meningeal lymphatic channels. Direct tracer injection and imaging yields unambiguous physical evidence of tracer movement from CSF into parenchyma and then into clearance pathways. Moreover, combining tracer molecular-weight (MW) panels (small vs. large tracers) allows testing of size-dependent transport and barrier filters. Finally, terminal histology provides cellular-level localization not possible with noninvasive imaging alone.

### Key pre-clinical empirical findings

Pre-clinical experimental work across rodent and large-animal models has generated the core empirical framework for understanding glymphatic influx, interstitial exchange, and lymphatic clearance, forming the mechanistic foundation for translational studies in humans [1–3].

- **Paravascular influx**: CSF enters the brain along peri-arterial spaces and exchanges with interstitial fluid, and was first visualized with fluorescently-labelled protein tracers [20].
- **Arterial pulsatility and sleep are major drivers**: Arterial pulsation enhances influx; sleep (or anesthesia) increases interstitial space and glymphatic transport, boosting clearance [21, 22]. The solute drainage is more active during deep sleep (NREM) and is impaired after sleep deprivation [23–25]. Ketamine-xylazine and dexmedetomidine increase the glymphatic solute influx (from SAS-CSF to PVS-CSF compartment), and potentially augment its drainage, while isoflurane inhibits the function [22, 26, 27].
- **Size dependence**: Small molecules penetrate deeper into ISF than large macromolecules; high-MW tracers show delayed or limited parenchymal distribution and preferential efflux via lymphatic and perineural sheath space routes [15].
- **Meningeal lymphatics and cervical node drainage**: Tracers injected into CSF drain to dural lymphatic vessels, perineural sheath spaces, nasopharyngeal lymphatic plexus including superficial and deep cervical nodes, a major efflux pathway bridging CNS and systemic immune-lymphatic systems [3].
- **Glymphatic regulation in disease states**: Animal models of AQP4-knock out, hypertension, stroke, meningitis, heart failure, neurodegeneration including TBI have been used to probe drivers as well as therapeutic strategies targeted towards glymphatic flux [4–7, 16, 17, 28, 29]. Interestingly, glymphatic failure can trigger early brain edema in model of ischemic stroke, cardiac arrest and TBI [4, 5, 7]. Rodent studies have ascertained an impairment in the glymphatic solute drainage along the cervical lymphatic vessels after TBI, which is rescued by a pan-adrenergic blockade [4]. Moreover, brain injury biomarkers such as GFAP, S100B and NSE are transported along the glymphatic system, with their drainage into peripheral blood being altered after TBI upon relevant glymphatic modulations (e.g., sleep deprivation, cisternotomy) [30].

Furthermore, the Miura et al. (2018) intraventricular vancomycin microdialysis model provides the first experimentally tractable framework for directly measuring solute transfer from CSF into brain extracellular space [31], thereby operationalizing the core premise of the microGLYMPH concept. By pairing CSF-based delivery with parenchymal concentration monitoring, this model establishes a quantifiable platform for investigating neurofluid exchange mechanisms, testing physiological modulators of glymphatic transport, and enabling ethically and technically feasible translation into human patients already undergoing ventricular drainage and CMD monitoring. Collectively, these pre-clinical findings define the essential features of glymphatic transport, such as peri-arterial influx, sleep and pulsatility dependency, molecular size constraints, and lymphatic outflow, while showing how injury and disease disrupt these pathways. Yet almost all evidence comes from tracer-based rodent studies that cannot be replicated in humans, thus microGLYMPH directly addresses this gap by translating these mechanistic signatures into a bedside, temporally resolved method for tracking human CSF–ISF solute exchange. It provides the first practical means to test, in patients, whether the hallmark behaviors of glymphatic flow observed in animals hold true after acute brain injury.

### Clinical

CMD is routinely used in specialized centers to monitor severe TBI and stroke patients and quantify interstitial brain metabolites (e.g., lactate, pyruvate, glucose, glutamate, glycerol) in conjunction with multimodal invasive and non-invasive neuromonitoring tools [12, 32, 33]. Macrocyclic gadobutrol or Gadavist (off-label), and iodinated contrast agent, iohexol or Omnipaque (FDA-approved), including radiotracers have been used in cisternography for detecting CSF leaks, and hydrocephalus assessment (Table 1). Intrathecal gadobutrol-based DCE-MRI safely investigated in patients, showed signal enhancement in the brain parenchyma as well as along the glymphatic inflow and outflow pathways such as periarterial SAS, parasagittal dural space, cervical lymph nodes including skull bone marrow [9, 11, 34–37]. Remarkably, human sleep deprivation impairs CSF solute clearance as assessed using intrathecal gadobutrol [38]. The contrast level in blood plasma [39] and/or urine can serve as a measure of glymphatic clearance (contrast egress from brain to the periphery). Importantly, intrathecal administration of GBCAs remains off‑label and carries regulatory safety warnings; where considered, it should be framed as research‑grade use with explicit risk mitigation and supported by published safety series, and iodinated intrathecal agents with labelled indications may be preferable for early feasibility work [13, 14, 40, 41].

### microGLYMPH as a human bridge to glymphatic translation

**Fig. 1:**
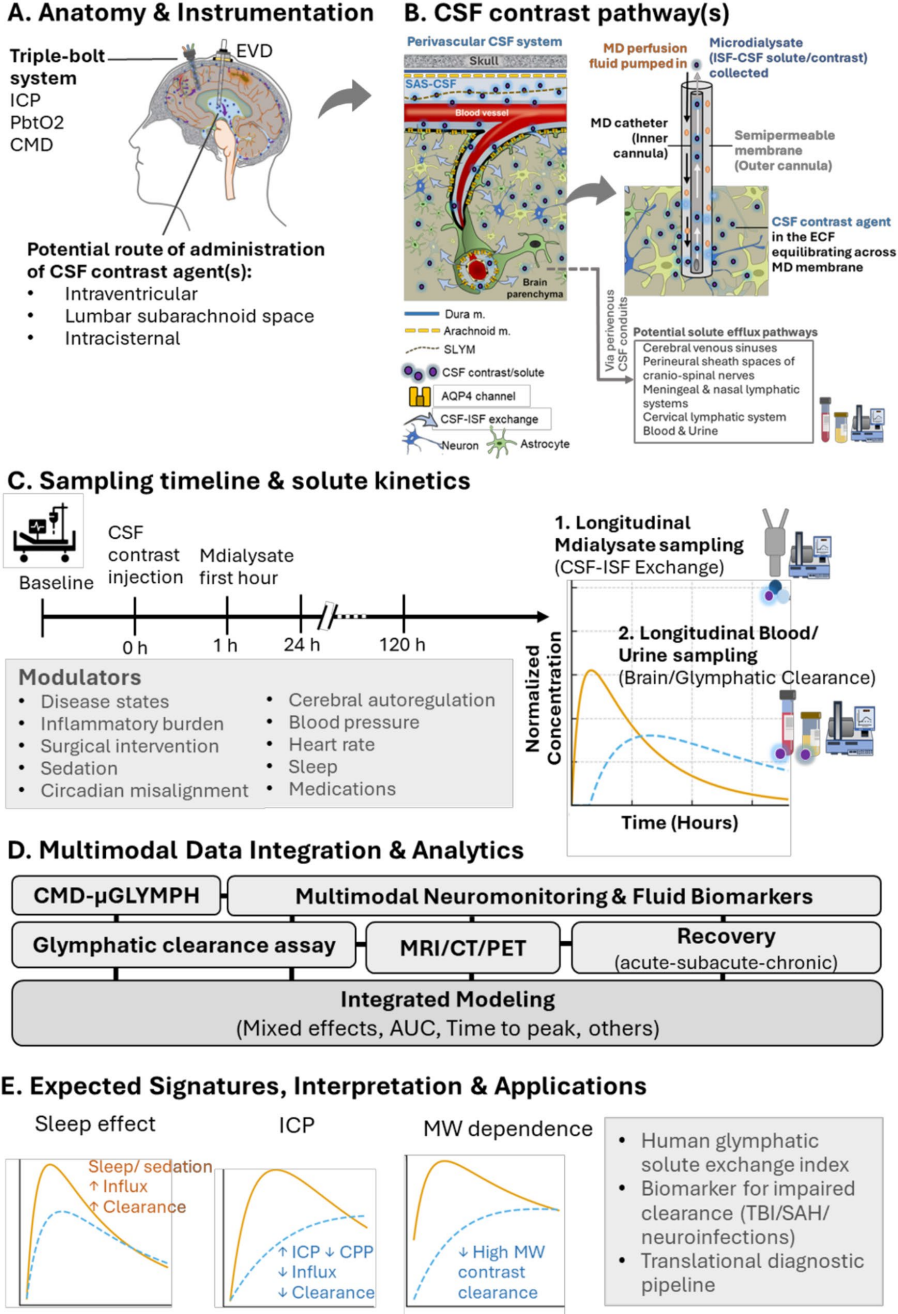
Overview of the microGLYMPH experimental and translational framework. This schematic illustrates the approach for quantifying glymphatic solute exchange and clearance in humans with acquired brain injury, by adapting the animal gold-standard tracer approach. This schematic illustrates the microGLYMPH approach for measuring glymphatic solute exchange in humans with acute brain injury. **(A)** Intracranial monitoring includes a triple-bolt for intracranial pressure (ICP), partial pressure of brain tissue (PbtO₂), and cerebral microdialysis (CMD), together with an external ventricular drain (EVD) enabling controlled intraventricular, intrathecal, or intracisternal administration of clinically approved tracers at diagnostic doses. **(B)** The tracer trajectory from CSF to interstitial fluid, perivascular pathways, and lymphatic/venous efflux is shown. **(C)** Longitudinal CMD sampling is integrated with blood and urine tracer kinetics and optional DCE-MRI. **(D)** Multimodal analysis combines microdialysate profiles with neuromonitoring and biomarkers. For instance, non-invasive monitoring (Doppler flow velocity, pulsatility indices, optic nerve sheath diameter, pupillometry, ICP waveform analysis, EEG) provides physiologic context. **(E)** Expected signatures include sleep-enhanced influx, ICP-related suppression, and molecular weight–dependent kinetics. By combining the above, microGLYMPH enables direct human assessment of glymphatic solute movement and clearance

Existing neurometabolic monitoring (CMD, or partial pressure of brain oxygenation, PbtO2) is not sufficient to resolve solute clearance mechanisms [33]. The proposed approach provides a transformative framework to quantify human glymphatic solute exchange in vivo, integrating temporal precision, physiological context, and translational feasibility within clinical neuromonitoring environments (Fig. 1). Key advantages include: (i) It provides the missing human in vivo time-series of ISF solute kinetics unmatched by imaging alone including conventional inherent proteins and neurometabolites such as lactate, pyruvate, glutamate, glucose, as well as CSF solute or tracer allowing a bidirectional evaluation of solute exchange (Fig. 1A, B). (ii) Mechanistic correlations by integrating neuromonitoring and testing mechanistic hypotheses derived from animals (arterial pulsatility and sleep effects) in patients with acute brain injury. (iii) Dual readout using bedside ISF kinetics along with systemic clearance (blood and/or urine) and/or optional MRI for spatial glymphatic mapping (Fig. 1C, D). Tracer clearance to blood/urine offers a peripheral biomarker of glymphatic function that could be used in future trials of glymphatic-modifying interventions. (iv) Ability to test size-dependence (low vs. high MW) and state-dependence (sleep, sedation, intracranial dynamics) of CSF-ISF exchange (Fig. 1E). (v) Safety and feasibility precedent considering minimal additional invasiveness for already-instrumented patients (leverages existing EVD, intracranial bolt, and microdialysis clinical placements) with clinical justification and ethical consideration.

### Proposed first‑in‑human operational pathway and feasibility endpoints

We emphasize that microGLYMPH is not yet a validated clinical platform; initial studies should therefore address feasibility rather than biomarker claims. A pragmatic first-in-human approach would be a narrowly defined cohort of already instrumented patients, such as those with severe TBI or poor-grade subarachnoid hemorrhage, in whom CMD and an EVD, or a lumbar drain or a cisternal catheter are placed for standard care. Tracer delivery should use existing access where possible, for example intraventricular administration via the EVD, with drainage settings and sampling times prespecified to limit confounding.

For CMD, feasibility reporting should specify catheter type, perfusion fluid, flow rate, sampling interval, and calibration or recovery assumptions. A flow rate of around 0.3 µL/min with hourly sampling is standard in neurocritical care and provides realistic temporal resolution. Quantification should specify the assay platform, for example ICP-MS, HPLC-UV, or LC-MS/MS, and include a minimal kinetic plan comprising concentration-time profiles, time to appearance, time to peak, area under the curve, and sensitivity analyses across drainage settings and injury severity [32, 42, 43]. Minimal feasibility endpoints include: (i) tracer detectability above the lower limit of quantification; (ii) tolerability and safety of the administration route; (iii) protocol adherence and sampling completeness; and (iv) interpretable kinetics under prespecified physiological conditions permitting comparison across individuals [13, 42].

### Disentangling true glymphatic transport from diffusion and injury-related leakage

A key challenge for human glymphatic assessment is distinguishing genuine CSF–ISF exchange from passive diffusion or tracer leakage related to blood-brain barrier (BBB) disruption or catheter-adjacent injury. microGLYMPH addresses this through temporally resolved microdialysate kinetics, size-selective tracers, and multimodal physiological context (Fig. 1). Glymphatic transport produces delayed, state-dependent ‘influx–peak–clearance’ profiles, whereas passive diffusion shows rapid early appearance and monotonic equilibration. Using both small and large tracers provides an internal control: large molecules that cannot freely diffuse but still appear with delayed, pulsatility-linked kinetics strongly indicate perivascular or lymphatic transport rather than leakage. Correlation with ICP, PbtO2, neurofluid dynamics (‘neurofluids’ comprise all fluid compartments in the brain and spine such as blood, CSF and ISF), and sleep/sedation state (EEG/Bispectral Index) further anchors tracer movement to physiological drivers of convective flow. Local tissue injury is accounted for by monitoring microdialysate brain injury (neural and astrocytic-specific) markers and by using structural MRI to map perilesional edema or necrosis. Finally, longitudinal systemic tracer detection in blood and urine provides external validation, as true CNS solute efflux should be reflected in peripheral clearance. By enforcing internally consistent kinetics across modalities and by validating that tracer appearance aligns with known glymphatic dependencies, including sleep, pulsatility, brain compliance and molecular weight, microGLYMPH establishes a robust interpretive framework capable of isolating physiologic glymphatic transport even in the inherently heterogeneous environment of acute neurological disease.

### Limitations

microGLYMPH has translational potential, but its limitations need to be made clear. CMD is inherently focal, and microdialysate kinetics therefore reflect regional CSF–interstitial tracer behavior rather than whole-brain “glymphatic function.” In acquired brain injury, tracer appearance may also be influenced by major confounders, including blood–brain barrier disruption, oedema, necrosis, hemorrhage, catheter effects, and CSF diversion, quite apart from any glymphatic-like transport. Patient heterogeneity should be accounted for from the outset, whether through control, stratification, or modelling. Peripheral tracer measurements in blood or urine may be informative, but they are non-specific, reflecting systemic pharmacokinetics and renal function as well as CNS efflux. The intrathecal use of some tracers, particularly GBCAs, also remains off-label and subject to safety warnings, which makes explicit ethical justification and staged feasibility safeguards essential. microGLYMPH is therefore best presented as an experimental translational approach that requires careful design, recovery verification, and cautious interpretation, especially given ongoing debate over fluid-transport pathways and the relative contributions of advection and diffusion in humans.

### Future directions

This work opens avenues for multifaceted exploration of glymphatic dynamics in health and disease for prognosis and putative therapeutics (Fig. 1). Future directions include: (i) Comparing solute exchange dynamics between different neurological states (e.g., coma vs. sedation vs. natural sleep). (ii) Combining CMD with imaging modalities (e.g., multimodal bedside neuromonitoring platform such as transcranial Doppler, automated pupillometry, ICP waveform analyzer, novel neurofluids coupling monitor, fluid-based brain injury biomarkers, DCE-MRI, recovery scales) for simultaneous structural-functional correlation. (iii) Testing therapeutic interventions (e.g., anti-edema measures, (hinge) craniotomy, decompressive craniectomy, Lund Concept, basal cisternostomy, adrenergic modulation, non-pharmacological brain acceleratory paradigms, sleep modulation) for glymphatic enhancement. (iv) Developing computational pharmacokinetic models of CSF-ISF-periphery exchange based on tracer kinetics.

## Conclusions

The conceptual microGLYMPH framework provides a mechanistically informed and clinically practical means of assessing surrogate measure of the glymphatic function, particularly, the CSF–ISF solute dynamics in humans. By leveraging CMD in neurosurgical patients, it enables real-time measurement of CSF–to–interstitial solute movement and offers a platform to examine how arousal state, intracranial dynamics, and vascular regulation shape glymphatic transport at the bedside. Clinically, microGLYMPH introduces a novel biomarker for detecting glymphatic dysfunction, in acute brain injury including TBI, intracranial bleeds and neuroinfections, wherein impaired clearance may contribute to secondary injury and chronic neurodegeneration. As a scalable, longitudinal tool, it has the potential to guide targeted interventions and support precision neurocritical care.

## Data Availability

No datasets were generated or analysed during the current study.

## Abbreviations

AQP4: Aquaporin-4
BBB: Blood-brain barrier
CMD: Cerebral microdialysis
CNS: Central nervous system
CSF: Cerebrospinal fluid
DCE-MRI: Dynamic contrast enhanced magnetic resonance imaging
EEG: Electroencephalography
HPLC-UV: High performance liquid chromatography - ultraviolet
ICP: Intracranial pressure
ICP-MS: Inductively coupled plasma-mass-spectrometry
ISF: Interstitial fluid
GBCAs: Gadolinium-based contrast agents
GFAP: Glial fibrillary acidic protein
MW: Molecular weight
NSE: Neuron-specific enolase
PbtO2: Partial pressure of brain oxygenation
PVS: Perivascular spaces
NREM: Non-rapid eye movement
S100B: S100 calcium-binding protein B
SAS: Subarachnoid space
SPECT-CT: Single photon emission computed tomography
^99m^Tc-DTPA: ^99m^Technitium - Diethylene triamine penta acetic acid
TBI: Traumatic brain injury
